# Clinical exome-based panel testing for medically actionable secondary findings in a cohort of 383 Italian participants

**DOI:** 10.3389/fgene.2022.956723

**Published:** 2022-11-10

**Authors:** Stefania Martone, Autilia Tommasina Buonagura, Roberta Marra, Barbara Eleni Rosato, Federica Del Giudice, Ferdinando Bonfiglio, Mario Capasso, Achille Iolascon, Immacolata Andolfo, Roberta Russo

**Affiliations:** ^1^ Dipartimento di Medicina Molecolare e Biotecnologie Mediche, Università Degli Studi di Napoli Federico II, Napoli, Italy; ^2^ CEINGE Biotecnologie Avanzate, Napoli, Italy; ^3^ Dipartimento di Ingegneria Chimica, Dei Materiali e Della Produzione Industriale, Università Degli Studi di Napoli Federico II, Napoli, Italy

**Keywords:** secondary findings, clinical exome, genetic testing, ACMG actionable genes, NGS testing

## Abstract

**Background:** Next-generation sequencing-based genetic testing represents a great opportunity to identify hereditary predispositions to specific pathological conditions and to promptly implement health surveillance or therapeutic protocols in case of disease. The term secondary finding refers to the active search for causative variants in genes associated with medically actionable conditions.

**Methods:** We evaluated 59 medically actionable ACMG genes using a targeted *in silico* analysis of clinical exome sequencing performed in 383 consecutive individuals referred to our Medical Genetics Unit. A three-tier classification system of SFs for assessing their clinical impact and supporting a decision-making process for reporting was established.

**Results:** We identified SFs with high/moderate evidence of pathogenicity in 7.0% (27/383) of analyzed subjects. Among these, 12/27 (44.4%) were carriers of a high-risk recessive disease allele. The most represented disease domains were cancer predisposition (33.3%), cardiac disorders (16.7%), and familial hypercholesterolemia (12.5%).

**Conclusion:** Although still debated, ensuring during NGS-based genetic testing an opportunistic screening might be valuable for personal and familial early management and surveillance of medically actionable disorders, the individual’s reproductive choices, and the prevalence assessment of underestimated hereditary genetic diseases.

## Introduction

Next-generation sequencing (NGS) technologies allow molecular analysis of several genes at the same time (targeted or extended panels), the set of DNA coding sequences (e.g., whole-exome), or the entire genome. These approaches increase the rate of diagnosis of rare diseases; however, they also provide information beyond the clinical question, finding variants in unexpected genes, defined as incidental or secondary findings. (Green et al., 2013) Particularly, the term secondary finding (SF) refers to the active search for causative variants in genes associated with medically actionable conditions. (Green et al., 2013) The use of NGS data could represent a great opportunity to recognize hereditary predispositions to specific pathological conditions and to promptly implement health surveillance or therapeutic protocols in case of the disease. However, the individual and even family psychological impact resulting from the knowledge of a genetic predisposition to a disease is subjective and unpredictable and it should not be overlooked. Therefore, it is debated whether to ensure during a DNA sequencing analysis an opportunistic screening test to deliberately research predispositions to hereditary genetic diseases or to identify the carriership of a recessive disease affecting an individual’s reproductive choices. (Burke et al., 2013; Brothers et al., 2019; Woudstra et al., 2021).

The American College of Medical Genetics (ACMG) recommended that SFs research should be applied as part of a clinical genetic test with diagnostic value to adults and minors, with an opt-out approach. (Green et al., 2013) Since 2013, ACMG periodically updates the list of actionable genes. Originally, a list of 57 actionable genes was proposed, subsequently reduced to 56, and after updated to 59 actionable genes. (Kalia et al., 2017) At the time of writing, this list has been expanded to 73 genes. (Miller et al., 2021a)

According to the concept of opportunistic screening, the European Society of Human Genetics (ESHG) recommended that SFs research should be carried out in the framework of pilot projects; it should only be applied to adults with rare exceptions in minors, with an opt-in approach and the selection of actionable genes should be contextual. Indeed, no actionable gene list was provided by ESHG. (van El et al., 2013; de Wert et al., 2021)

In this study, we aim to assess the frequency of SFs in a case series of 383 consecutive Italian patients referred to our Medical Genetics diagnostic Unit of CEINGE Institute from November 2020 to September 2021. We focused on 59 actionable ACMG genes (Kalia et al., 2017) by a targeted *in silico* analysis of clinical exome sequencing data. Overall, we identified SFs with high/moderate evidence of pathogenicity in 7.0% of the analyzed subjects. As a second purpose, we herein proposed a three-tier classification system of SFs to support a decision-making process for reporting.

## Materials and methods

### Patients enrolled in this study and genomic DNA preparation

Overall, 383 consecutive subjects referred to our Medical Genetics diagnostic Unit of CEINGE Institute from November 2020 to September 2021 were included in this study. They were analyzed by clinical exome after genetic counseling.

The case series includes 266 unrelated families, 284 subjects were index cases, and 99 were first-degree relatives (parents and/or siblings). One hundred and fifty-six subjects were pediatrics (0–18 years), whereas 227 were adults (19–75 years). One hundred and ninety-three were females (50.4%), and 190 were males (49.6%).

DNA samples were obtained from each adult subject after signed informed consent, and according to the Declaration of Helsinki. Written informed consent was provided by the participants’ legal guardian/next of kin for pediatric subjects. Prior informed consent to explain the advantages and limits of opportunistic genomic screening has been obtained, allowing the communication of secondary findings exclusively to adult enrolled patients, with an opt-in approach as recommended by ESHG guidelines. Subsequently, we reviewed the patient on the delivery of the original report and we investigated the family history (“dynamic” consensus). This approach was used mainly in the case of the identification of SFs with moderate evidence of pathogenicity.

Genomic DNA preparation was performed using the Maxwell RSC Blood DNA Kit for automated extraction (Promega, Milan, Italy). To evaluate the quality of the extracted genomic DNA before fragmentation, samples were quantified using a UV-Vis spectrophotometer (NanoDrop 2000; Thermo Scientific, Waltham, MA, United States). Then, the genomic DNA was run on 0.8% agarose DNA gel electrophoresis.

### Sequencing and data analysis

Genetic testing has been performed by clinical exome, i.e., a test that looks at the protein-coding regions of the genome that have a known clinical association with diseases. In particular, we used a commercially available panel composed of >5000 genes associated with hereditary diseases (SureSelect custom Constitutional Panel 17 Mb, Agilent Technologies). Sample preparation was performed using the Illumina platform’s target enrichment (SureSelectQXT, Agilent Technologies) according to the manufacturer’s instructions. High-throughput sequencing was performed using an Illumina NextSeq 500 platform. The alignment of sequencing reads to the genomic reference, quality control, and identification of variants were carried out with Alissa Align and Call software (v1.1.3–4; Agilent Technologies). Reads were aligned to the GRCh37/hg19 version of the human genome using the BWA MEM algorithm, exons and regions with a read depth below 20 were discarded. Variant annotation and analysis were performed using the Alissa Interpret software (v5.2.10; Agilent Technologies).

The opportunistic genomic screening was performed using a targeted *in silico* panel (OGS panel) composed of 59 medically actionable genes listed in the ACMG guidelines (Supplementary Table S1). (Kalia et al., 2017) The prioritization of each SF was obtained by gathering evidence from various sources: population data, computational, and pathogenicity scores. Data were filtered for variants present in less than 1% gnomAD, ExAC, and 1000Genomes populations. Rare variants with a frequency >1% in the tested cohort (mainly indels located in regions with low mappability or in homopolymeric regions) were further excluded. In agreement with the definition of screening testing, the prioritized SFs were not confirmed by Sanger sequencing. Nevertheless, read depth (>20), alternative allele frequency (>0.25), and visual inspection of the alignment were evaluated for each variant.

### Secondary findings interpretation and classification

All prioritized SFs were firstly analyzed by ClinVar, a public repository that reports the correlation between genetic variants and phenotypes, with supporting evidence, to select pathogenic or likely pathogenic (PLP) SFs. Each variant was manually classified according to the current ACMG guidelines for variant interpretation. (Richards et al., 2015)

Based on both ClinVar interpretation and ACMG classification of the identified SFs, we generated a three-tier classification system of prioritized SFs (Table 1). The first and the second tier include SFs with high evidence of pathogenicity, i.e., either PLP ClinVar variants classified as PLP by ACMG or ClinVar variants with conflicting interpretations of pathogenicity (CI) with PLP interpretations ≥80% and classified as PLP by ACMG guidelines. Finally, the third tier includes SFs with moderate evidence of pathogenicity, i.e., either ClinVar variants with CI or variants not reported in ClinVar, but classified as PLP by ACMG.

**TABLE 1:** Classification criteria of secondary findings.

Class	Description	Evidence Level	Implication for Reporting^a^
Class I	- ClinVar PLP variants classified as PLP or VUS-LP by ACMG	High	To be reported
Class II	- ClinVar CI variants with PLP submitted interpretations >80% and predicted as PLP by ACMG	High	To be reported
Class III	- ClinVar CI variants with PLP submitted interpretations ≥50% and predicted as PLP by ACMG	Moderate	To be reported only after careful evaluation of the family history
	- ClinVar NR variants predicted as PLP by ACMG		

^a^ Secondary findings with high evidence are recommended to be reported after opt-in informed consent; secondary findings with moderate evidence are suggested to be reported after opt-in informed consent only in presence of positive family history.

ACMG, american college of medical genetics; CI, conflicting interpretations of pathogenicity; NR, not reported; PLP, pathogenic and likely pathogenic; VUS, variant of uncertain significance; VUS-LP, VUS-favor pathogenic. ClinVar, https://www.ncbi.nlm.nih.gov/clinvar/

## Results

### Description of secondary findings identified in the cohort of patients

**FIGURE 1:**
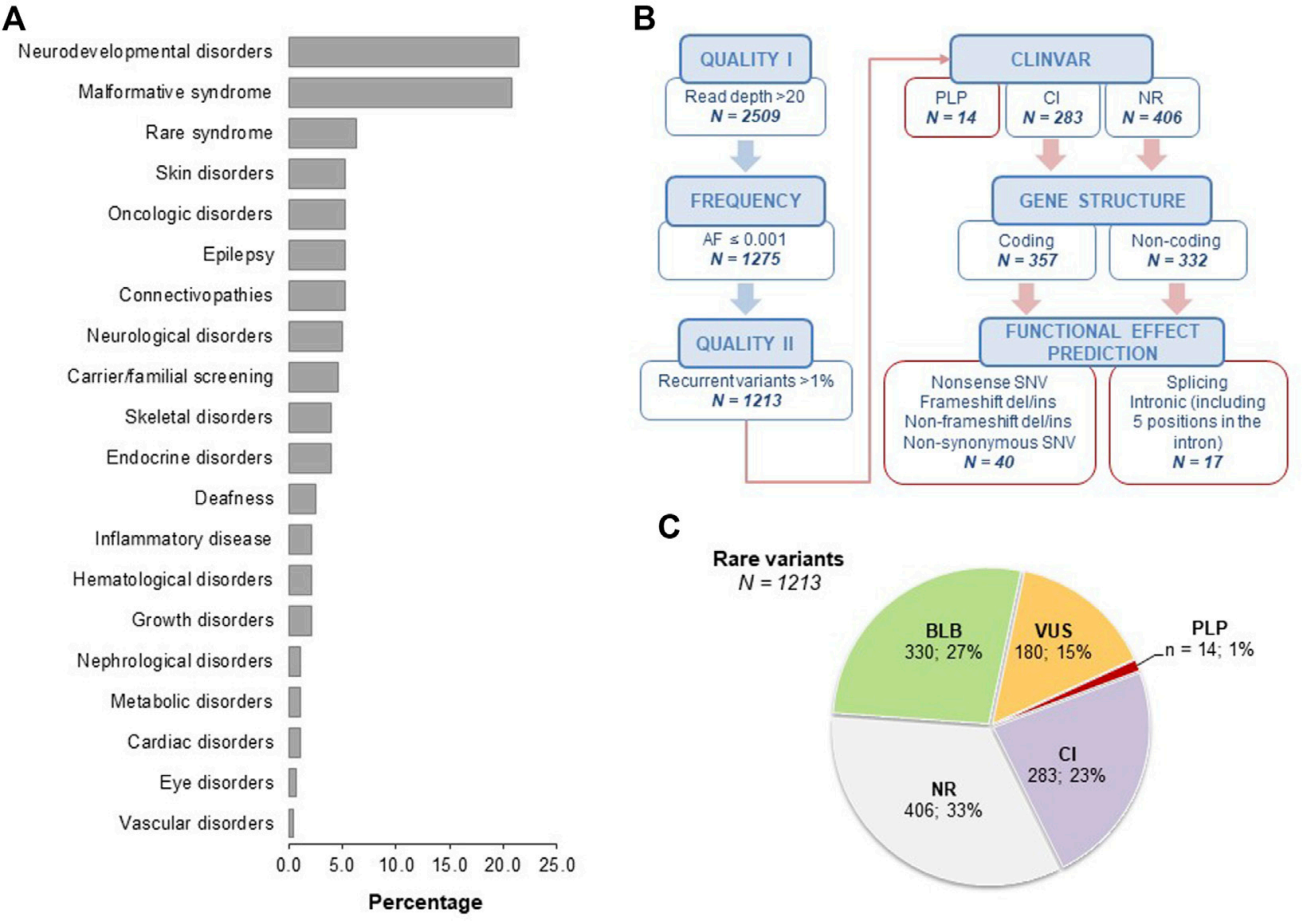
Analysis of case series and secondary findings. Panel **(A)**. Clinical indications of the 284 index cases enrolled in the study, subdivided as neurodevelopmental disorders (21.5%), malformative syndromes (20.8%), rare syndrome (6.3%), skin disorders (5.23%), oncologic disorders (5.3%), epilepsy (5.3%), connectivopathies (5.3%), neurological disorders (4.9%), carrier/familial screening (4.6%), skeletal disorders (3.9%), endocrine disorders (3.9%), deafness (2.5%), inflammatory disease (2.1%), hematological disorders (2.1%), growth disorders (2.1%), nephrological disorders (1.1%), metabolic disorders (1.1%), cardiac disorders (1.1%), eye disorders (0.7%), vascular disorders (0.4%). Panel **(B)**. Schematic representation of the filtering strategy for rare SF. The frequency filter was based on data retrieved from GnomAD_exome, GnomAD_genome, ExAC, and 1000Genomes population databases. Recurrent rare variants with frequency >1% in the tested cohort were further excluded. For coding nonsynonymous variants, data were filtered based on the agreement among at least 6/7 prediction tools (CADD Phred >20, LRT: D, MutationAssessor: H-M, MutationTaster: A-D, PolyPhen2 HumDiv: D-P, SIFT: D, FATHMM: D). For noncoding variants, we included essential splice donor/acceptor variants and intronic variants within five positions in the intron. AF, alternative allele frequency; PLP, pathogenic and likely pathogenic variants; CI, conflicting interpretation; NR, not reported; SNV, single nucleotide variant. Panel **(C)**. Pie charts showing the classification of rare SFs as obtained from ClinVar. BLB, benign/likely benign; PLP, pathogenic/likely pathogenic; VUS, variants of uncertain significance; CI, conflicting interpretation; NR, not reported.

Within our case series, the most common indications for genetic testing were neurodevelopmental disorders (61/284 index cases, 21.5%) and malformative syndromes (59/284 index cases, 20.8%) (Figure 1A).

The average sequencing depth was 142.2 with 98.5% of target regions at 20× coverage and 96.4% at 50×. Among the subjects analyzed by the OGS panel (n = 383), we overall identified 1213 rare variants, on average 3.2 rare variants for each subject (Figure 1B). In particular, 14 out of 1213 variants (1%) were annotated in ClinVar as PLP, 23% (283/1213) as CI, while 33% (406/1213) were not reported (NR) (Figures 1B,C). Based on gene structure and functional effect prediction, we prioritized 40 ClinVar CI variants and 17 ClinVar NR variants (Figure 1B; Supplementary Tables 2–4). Following manual ACMG classification, we identified eight PLP SFs (7 from the ClinVar CI group and one from the ClinVar NR group). Thus, our filtering strategy enabled us to detect 21 unique SFs in 27 subjects (22 unrelated), with a range of 1-2 prioritized SFs for each subject (Table 2). All the identified variants were in the heterozygous state. We detected a single variant for each subject except for one subject who carried two variants.

**TABLE 2:** Clinical exome medically actionable secondary findings identified in 383 participants.

Gene	HGVS	HGVS	RefSeq ID	AF^a^	ClinVar° (N Submissions)	HGMD^b^	ACMG Classification
	cDNA-Level Nomenclature	Protein-Level					
Class I							
ATP7B	NM_000053.4:c.2906G>A	p.Arg969Gln	rs121907996	T=0.000036	P (8)	CM950116	LP
							PM1,PM2,PM5,PP2,PP3,PP5
ATP7B	NM_000053.4:c.2519C>T	p.Pro840Leu	rs768671894	-	P (8)	CM980172	LP
							PM2,PM5,PP2,PP3,PP5
ATP7B	NM_000053.4:c.51+4A>T	-	rs369488210	A=0.000016	P (10)	CS094248	VUS-LP
							PM2,PP3,PP5
DSP	NM_004415.4:c.4198C>T	p.Arg1400*	rs770873593	T=0.000004	PLP (6)	CM1312989	P
							PVS1,PM2,PP5
FBN1	NM_000138.5:c.6739+1G>A	-	rs869025419	-	PLP (2)	CS075143	P
							PVS1,PM2,PP5
KCNH2	NM_000238.4:c.1129–2A>G	-	rs794728365	-	P (2)	CS002454	P
							PVS1,PM2,PP5
LDLR	NM_000527.5:c.465C>A	p.Cys155*	rs766094434	A=0.000004	P (4)	CM011395	P
							PVS1,PM2,PP5
LDLR	NM_000527.5:c.1775G>A	p.Gly592Glu	rs137929307	A=0.000044	P (36)	CM920464	P
							PS3,PM1,PM2,PM5,PP2,PP3,PP5
MSH6	NM_000179.3:c.3514dupA	p.Arg1172Lysfs*5	rs63751327	dupA=0.000008	P (12)	CI992289	P
							PVS1,PM2,PP5
MUTYH	NM_001048174.2:c.1103G>A	p.Gly368Asp	rs36053993	T=0.003027	PLP (64)	CM020287	P
							PS3,PM2,PM5,PP3,PP5
MUTYH	NM_001048174.2:c.228C>A	p.Tyr76*	rs121908380	-	P (12)	CM022646	P
							PVS1,PM2,PP5
PTEN	NM_000314.8:c.475A>G	p.Arg159Gly	rs786202688	-	LP (3)	CM110139	LP
							PM1,PM2,PM5,PP2,PP3,PP5
RET	NM_020975.6:c.2410G>A	p.Val804Met	rs79658334	-	PLP (25)	CM981707	LP
							PM2,PM5,PP2,PP3,PP5
RYR1	NM_000540.3:c.1841G>A	p.Arg614His	rs193922772	A=0.00002	LP (2)	-	LP
							PM2,PM5,PP3,PP5
Class II							
ATP7B	NM_000053.4:c.2605G>A	p.Gly869Arg	rs191312027	-	CI	CM052169	LP
					VUS(1),LP (7),P (10)		PM1,PM2,PM5,PP2,PP3,PP5
LDLR	NM_000527.5:c.1003G>A	p.Gly335Ser	rs544453230	-	CI	CM920439	LP
					LB (1),VUS(1),LP (8),P (2)		PM1,PM2,PM5,PP2,PP3,PP5
MUTYH	NM_001048174.2:c.849+3A>C	-	rs587780751	G=0.000076	CI	CS031781	VUS-LP
					VUS(1),P (17)		PM2,PP3,PP5
Class III							
ACTA2	NM_001613.4:c.554G>A	p.Arg185Gln	rs1057521105	-	CI	CM092903	LP
					VUS(1),LP (1)		PM1,PM2,PP2,PP3
ATP7B	NM_000053.4:c.19_20delCA	p.Gln7Aspfs*14	rs749363958	delGT=0.000152	CI	CD054306	P
					VUS(4),LP (3),P (1)		PVS1,PM2,PP5
MYBPC3	NM_000256.3:2429G>A	P.Arg810His	rs375675796	T=0.000048	CI	CM034546	LP
					VUS(3),LP (5)		PM1,PM2,PM5,PP3,PP5
DSP	NM_004415.4:c.6202_6205del	p.Thr2068Serfs*5	-	-	-	-	LP
							PVS1,PM2

All the identified variants are in the heterozygous state.

^a^ AF, alternative allele frequency as retrieved from GnomAD_exome.

°ClinVar PLP interpretations with ≥2 submissions.

^b^ HGMD Professional 2022.1. CI, conflict of interpretation; LB, likely benign; LP, likely pathogenic; P, pathogenic; VUS, variant of uncertain significance; VUS-LP, VUS-favor pathogenic.

To select SFs highly likely to be causative, we applied a three-tier classification system to prioritize SFs according to different evidence levels of pathogenicity (Table 1). Based on this classification, we observed SFs with high/moderate evidence of pathogenicity in 7.0% of analyzed subjects (27/383) and 8.3% of unrelated families (22/266). In particular, SFs with high evidence of pathogenicity (tiers I-II) were found in 6.0% of analyzed subjects (23/383) and 7.1% of unrelated families (19/266). Whereas SFs with moderate evidence of pathogenicity (tier III) were identified in 1.0% of analyzed subjects (4/383) and 1.5% of unrelated families (4/266).

### Molecular and clinical classification of secondary findings

**FIGURE 2:**
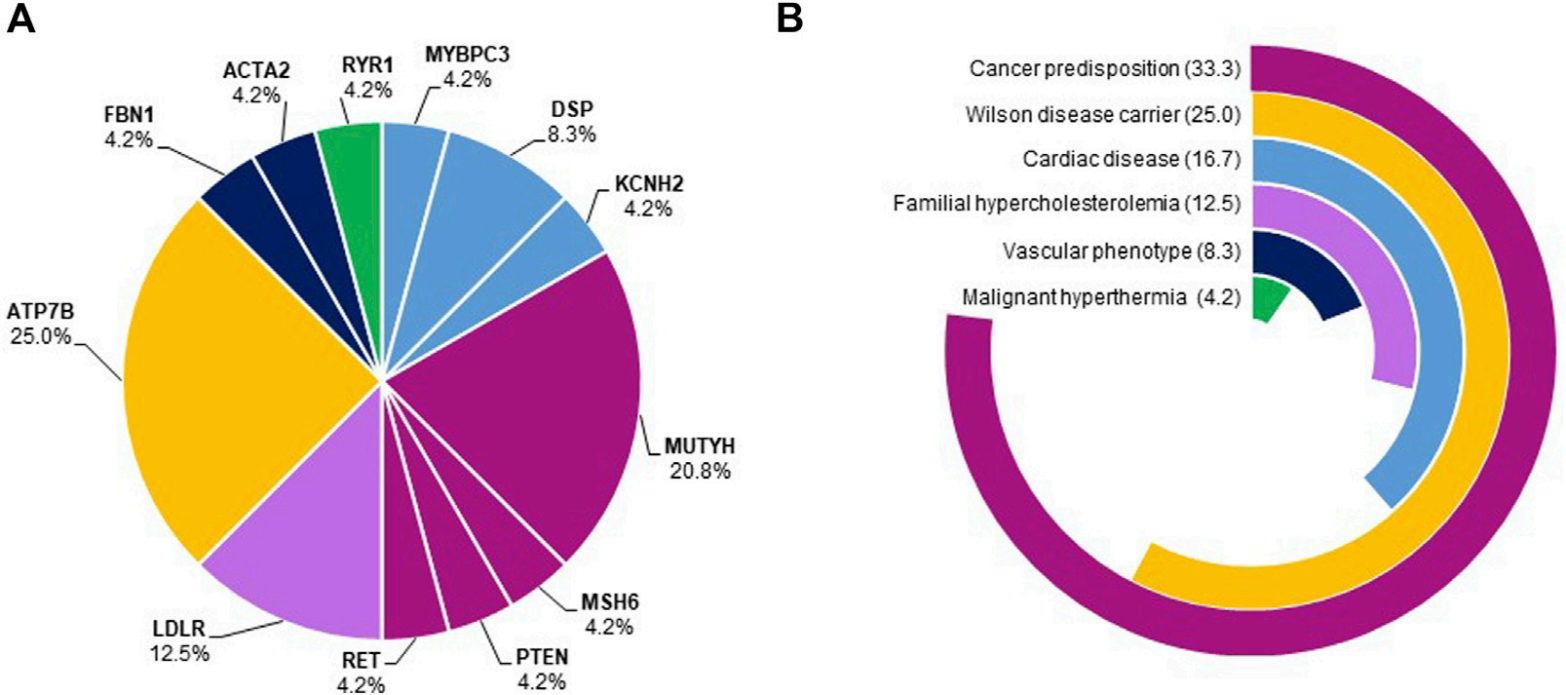
Frequency of genes harboring secondary findings and disease domains. Panel **(A)**. The pie chart shows the frequency of genes harboring SFs. Genes belonging to the same disease domain were shown using the color code reported in panel B. Panel **(B)**. The pie chart shows the proportions of disease domains of identified SFs. The most frequent disease domains were cancer predisposition (33.3%), cardiac diseases (16.7%), and familial hypercholesterolemia (12.5%).

Most of the subjects presented dominant medically actionable variants (15/27, 55.5%). The most recurrent genes harboring SFs with high/moderate levels of pathogenic evidence were *LDLR* and *DSP* (Figure 2A). Besides, 12 subjects carried at least one high-risk recessive allele (12/27, 44.4%) either in *ATP7B* or in *MUTYH* gene, causative of Wilson disease and *MUTYH*-associated polyposis, respectively (Figure 2A).

Of note, 33.3% of SFs with high/moderate evidence of pathogenicity were identified in loci related to cancer predisposition (*MUTYH*-associated polyposis, Lynch syndrome, multiple endocrine neoplasia type 2, Cowden syndrome), 16.7% in genes causative of cardiac disorders (arrhythmogenic right ventricular dysplasia, dilated cardiomyopathy, long QT syndrome), and 12.5% in genes associated with familial hypercholesterolemia (Figure 2B).

## Discussion

Secondary findings arise from the active search for causative variants in genes associated with medically actionable conditions. To date, it is debated whether to ensure, during NGS-based genetic testing, an opportunistic screening to research deliberately predispositions to hereditary genetic diseases or identify a state of carrier for recessive disorders affecting the individual’s reproductive choices. (Woudstra et al., 2021)

Using an *in silico* targeting of 59 medically actionable genes, we investigated SFs in a monocentric case series of 383 consecutive subjects undergoing a clinical exome in suspicion of different genetic conditions, mainly neurodevelopmental disorders, and malformative syndromes. Our study allowed us to detect overall 21 unique actionable SFs with high/moderate evidence of pathogenicity in 7.0% of analyzed subjects, 17 of them classified as SFs with high evidence of pathogenicity in 6.0% of individuals, in line with reported SFs frequencies ranging between 1.2% and 11%. (Xue et al., 2012; Dorschner et al., 2013; Cassa et al., 2013; Olfson et al., 2015; Jang et al., 2015; eMERGE Clinical Annotation Working Group, 2020; Jalkh et al., 2020; Aloraini et al., 2022) Different factors including study design, sequencing technology, tested population, the knowledge of variants in literature, and data interpretation explain the different rates of SFs reported in previous studies. Data interpretation is a complicated phase of the study of a variant, and the choice of a specific methodology may underestimate or overestimate the finding. We herein established a three-class ranking to prioritize secondary events based on their annotation/prediction using the ClinVar database and ACMG guidelines. (Richards et al., 2015) Through this approach, we tried to provide a clinically-driven method for assessing the impact of medically actionable genetic variations with direct implications on the choice to report or not a specific SF. In particular, we suggested that SFs with high evidence of pathogenicity (i.e., those included in the first two tiers) should be recommended for reporting in adult subjects after signing their opt-in informed consent regardless of information about their family history. Conversely, those variants with moderate evidence of pathogenicity (i.e., those included in the third tier) should be carefully re-evaluated considering the family history before inclusion in the genetic testing report. In these specific cases, the collection of family history through a “dynamic” consensus system as proposed by the SFMPP could be the right approach to reanalyze SFs with uncertain pathogenicity, in order not to miss SFs. (Pujol et al., 2018) The three-tier classification highlighted the difficulty of the molecular geneticists to report those SFs with moderate evidence of pathogenicity (tier III). Only the knowledge of positive family history for that specific disease can overcome the problem of reporting those SFs included in tier III. Of note, the current recommendations for reporting of SFs in clinical exome establish that all variants that are classified as P or LP according to ACMG/AMP standards and guidelines should be reported as SFs. (Miller et al., 2021b)

In agreement with previous similar studies, we identified SFs in genes mostly associated with cardiac disorders (16.7%), cancer predisposition (33.3%), and familial hypercholesterolemia (12.5%). (eMERGE Clinical Annotation Working Group, 2020; Aloraini et al., 2022; Ramensky et al., 2021) These findings underline the importance of establishing rapid and early screening methods to avoid the underestimation of medically actionable disorders.

In Southern Italy, the prevalence of hypertrophic cardiomyopathy, Lynch syndrome, or familial hypercholesterolemia is not well-established since prevalence studies are mainly based on screening in young adults and therefore biased by age-related penetrance. Similarly, incomplete penetrance may account for underestimated prevalence, as in the case of some mismatch repair genes causative of Lynch syndrome.

Based on the identified SF, the clinicians might suggest extending the communication to first-degree family members and eventually performing a single-variant diagnostic test on the relatives. Indeed, according to a multicenter study, the return of SFs in apparently healthy family members increases the diagnosis of hereditary conditions. (Hart et al., 2019) In parallel, clinicians should start monitoring the condition with adequate clinical and/or biochemical testing. Our data confirm the importance of detecting underdiagnosed disorders, such as familial hypercholesterolemia, especially in those countries where clinical scores are lacking, such as Italy, in contrast to those that routinely use clinical scores for diagnosis, such as the DUTCH score. (Nordestgaard et al., 2013; Bertolini et al., 2017; Reeskamp et al., 2020) Additionally, they also underline the relevance of identifying susceptibility to disorders for which the risk-to-benefit ratio of the recommended interventions favors such interventions since they are not overly burdensome or costly. This is the case of malignant hyperthermia susceptibility whose clinical diagnostic test is invasive and expensive. Nevertheless, most surgical procedures can be performed by avoiding known triggering agents, and effective measures can be taken if knowledge of the risk is communicated to the anesthesiologist. (Katz et al., 2020)

We identified 12 individuals with a recessive pathogenic disease allele in our case series. Reporting carrier status (e.g., in *ATP7B* gene) might be highly beneficial, especially in a population characterized by a high rate of consanguineous marriages for the recurrence risk assessment and valuable for the carrier itself. Indeed, it is well-known that heterozygous carriers of *MUTYH* alleles have an increased risk of colorectal cancer and *MUTYH* carriership is associated with younger age of diagnosis, and a higher prevalence of polyps, right-sided and synchronous cancers.

The return of genomic SFs in NGS testing represents a point of discussion among molecular and clinical geneticists. Our study estimated the frequency of clinically actionable SFs in a cohort of Italian patients. We herein provided a classification system of SFs to assess their clinical impact and guide the reporting, focusing on a manual clinically-driven approach to study each variant, and adopting a dynamic consensus.

The establishment of a three-tier ranking of prioritized SFs might facilitate the reporting of these variants thus significantly impacting patients’ follow-up and management. Our data showed that most medically actionable variants were in genes associated with cardiac disorders, oncological predisposition, and familial hypercholesterolemia, conditions that are probably underestimated. With the report of these findings, the patients will benefit from a multi-disciplinary approach, including genetic counseling, involving a detailed personal and family history, surveillance of the disease, and family plan.

## Data Availability

Aggregated variant frequency information can be requested to the corresponding author. Individual genotype information cannot be made available in order to protect participant privacy.
