# Supplementary material for: Clinical exome-based panel testing for medically actionable secondary findings in a cohort of 383 Italian participants
Source: Front Genet. 2022 Nov 10;13:956723. doi: 10.3389/fgene.2022.956723 (PMC9685519; doi:10.3389/fgene.2022.956723)
Supplement: Supplementary file 3 [file Table1.docx]

**Supplementary Data**

**Clinical exome-based panel testing for medically actionable secondary findings in a cohort of 383 Italian participants**

Stefania Martone^1,2,§^, Autilia Tommasina Buonagura^1,2,§^, Roberta Marra^1,2^, Barbara Eleni Rosato^1,2^, Federica Del Giudice^2^, Ferdinando Bonfiglio^2,3^, Mario Capasso^1,2^, Achille Iolascon^1,2^, Immacolata Andolfo^1,2,*^ and Roberta Russo^1,2,*^

^1^ Dipartimento di Medicina Molecolare e Biotecnologie Mediche, Università degli Studi di Napoli Federico II, Napoli, Italy

^2^ CEINGE Biotecnologie Avanzate, Napoli, Italy

^3^ Dipartimento di Ingegneria Chimica, dei Materiali e della Produzione Industriale, Università degli Studi di Napoli Federico II, Napoli, Italy

^§^ These authors equally contributed

***Correspondence:**

Roberta Russo, PhD

Assistant Professor of Medical Genetics

Dip. Medicina Molecolare e Biotecnologie Mediche

Università degli Studi di Napoli "Federico II"

CEINGE - Biotecnologie Avanzate

email: roberta.russo@unina.it

Immacolata Andolfo, PhD

Assistant Professor of Medical Genetics

Dip. Medicina Molecolare e Biotecnologie Mediche

Università degli Studi di Napoli "Federico II"

CEINGE - Biotecnologie Avanzate

email: immacolata.andolfo@unina.it

**Summary of content:**

- Supplemental Table 1. ACMG actionable genes

- Supplemental Table 2. Filtered PLP ClinVar variants

- Supplemental Table 3. Filtered CI ClinVar variants

- Supplemental Table 4. Filtered NR ClinVar variants

**Supplemental Table 1. ACMG actionable genes^§^**

| **Phenotype** | **Penetrance** | **OMIM** | **Gene** | **Inheritance** | **Typical age of onset** |  |
| --- | --- | --- | --- | --- | --- | --- |
| **Ornithine transcarbamylase deficiency** | Complete penetrance in hemizygous males | 311250 | *OTC* | XL | Male: newborn  Female: childhood/adulthood |  |
| **Retinoblastoma** | Complete penetrance (>99%) (null alleles); incomplete penetrance (≤25%) (in-frame, missense, splice site variants, certain indels in exon 1, or variants in the promoter region) | 180200 | *RB1* | AD | Childhood |  |
| **Tuberous sclerosis** | High penetrance | 191100 | *TSC1* | AD | Childhood |  |
|  |  | 613254 | *TSC2* |  |  |  |
| **Wilms tumor** | High penetrance (age-dependent, paternal origin) | 194070 | *WT1* | AD | Childhood |  |
| **Wilson disease** | Heterozygotes may have low serum ceruloplasmin concentrations, borderline normal urinary copper, elevated urinary copper on provocative testing with D-penicillamine, and/or moderate elevation of hepatic copper (100-250 mg/g dry weight) | 277900 | *ATP7B* | AR | Childhood/Adulthood |  |
| **Familial hypercholesterolemia** | Incomplete penetrance in heterozygous state | 144010 | *APOB* | SD | Childhood/Adulthood |  |
|  | 73% of heterozygous individuals have LDL level >130 mg/dL | 143890 | *LDLR* | SD | Childhood/Adulthood |  |
|  | High penetrance for p.Ser127Arg and p.Asp374Tyr variants | 603776 | *PCSK9* | AD | Childhood/Adulthood |  |
| **Malignant hyperthermia susceptibility** | Low or moderate penetrance | 145600 | *RYR1* | AD | Childhood/Adulthood |  |
|  |  | 601887 | *CACNA1S* |  |  |  |
| **Aortic aneurysm, familial thoracic** | Low or moderate penetrance | 611788 | *ACTA2* | AD | Childhood/Adulthood |  |
|  |  | 132900 | *MYH11* |  |  |  |
| **Arrhythmogenic right ventricular cardiomyopathy** | Low or moderate penetrance | 607450 | *TMEM43* | AD | Childhood/Adulthood |  |
|  |  | 604400 | *DSP* |  |  |  |
|  |  | 609040 | *PKP2* |  |  |  |
|  |  | 610193 | *DSG2* |  |  |  |
|  |  | 610476 | *DSC2* |  |  |  |
| **Hereditary breast and ovarian cancer** | Low or moderate penetrance. Females with pathogenic variants have up to 87% risk of developing associated cancer, while males have up to a 20% risk | 604370 | *BRCA1* | AD | Adulthood |  |
|  |  | 612555 | *BRCA2* |  |  |  |
| **Catecholaminergic polymorphic ventricular tachycardia** | Low or moderate penetrance | 604772 | *RYR2* | AD | - |  |
|  |  |  |  |  |  |  |
|  |  |  |  |  |  |  |
|  |  |  |  |  |  |  |
|  |  |  |  |  |  |  |
| **Supplemental Table 1**. ACMG actionable genes *(Continued)* | | | | | | |
| **Phenotype** | **Penetrance** | **OMIM** | **Gene** | **Inheritance** | **Typical age of onset** |  |
| **Familial hypertrophic cardiomyopathy, dilated cardiomyopathy** | Low or moderate penetrance | 192600 | *MYH7* | AD | Childhood/Adulthood |  |
|  |  | 115196 | *TPM1* |  |  |  |
|  |  | 115197 | *MYBPC3* |  |  |  |
|  |  | 600858 | *PRKAG2* |  |  |  |
|  |  | 613690 | *TNNI3* |  |  |  |
|  |  | 608751 | *MYL3* |  |  |  |
|  |  | 608758 | *MYL2* |  |  |  |
|  |  | 612098 | *ACTC1* |  |  |  |
|  |  | 301500 | *GLA* |  |  |  |
|  |  | 115200 | *LMNA* |  |  |  |
|  |  | 601494 | *TNNT2* |  |  |  |
| **Long QT syndrome (LQTS), Brugada syndrome** | Low or moderate penetrance | 192500 | *KCNQ1* | AD | Childhood/Adulthood |  |
|  |  | 613688 | *KCNH2* |  |  |  |
|  |  | 603830 | *SCN5A* |  |  |  |
| **Lynch syndrome** | Low or moderate penetrance  *MSH6* and *PMS2* variants: lower disease penetrance and older ages of diagnosis (colon cancer) | 609310 | *MLH1* | AD | Adulthood |  |
|  |  | 120435 | *MSH2* |  |  |  |
|  |  | 614350 | *MSH6* |  |  |  |
|  |  | 614337 | *PMS2* |  |  |  |
| **Paraganglioma-pheochromocytoma syndrome** | Low or moderate penetrance (age-related) | 168000 | *SDHD* | AD | Childhood/Adulthood |  |
|  |  | 601650 | *SDHAF2* |  |  |  |
|  |  | 605373 | *SDHC* |  |  |  |
|  |  | 115310 | *SDHB* |  |  |  |
| **Familial adenomatous polyposis** | High or near-complete penetrance | 175100 | *APC* | AD | Childhood/Adulthood |  |
| **Ehlers-Danlos syndrome, vascular type** | High or near-complete penetrance | 130050 | *COL3A1* | AD | Childhood/Adulthood |  |
| **Juvenile polyposis syndrome** | High or near-complete penetrance | 174900 | *BMPR1A* | AD | Childhood/Adulthood |  |
|  |  | 174900 | *SMAD4* |  |  |  |
| **Li-Fraumeni syndrome (LFS)** | High or near complete penetrance  Male: 70% or higher; female: 90% or higher | 151623 | *TP53* | AD | Childhood/Adulthood |  |
| **Loeys-Dietz syndrome, Familial thoracic aortic aneurysm and aortic dissection** | High or near-complete penetrance | 609192 | *TGFBR1* | AD | Childhood/Adulthood |  |
|  |  | 610168 | *TGFBR2* |  |  |  |
|  |  | 613795 | *SMAD3* |  |  |  |
|  |  |  |  |  |  |  |
|  |  |  |  |  |  |  |
| **Supplemental Table 1**. ACMG actionable genes *(Continued)* | | | | | | |
| **Phenotype** | **Penetrance** | **OMIM** | **Gene** | **Inheritance** | **Typical age of onset** |  |
| **Marfan syndrome** | High or near-complete penetrance  Variable intra-familial expressivity | 154700 | *FBN1* | AD | Childhood/Adulthood |  |
| **Multiple endocrine neoplasia, type 1** | High or near complete penetrance (age-related) | 131100 | *MEN1* | AD | Childhood/Adulthood |  |
| **Multiple endocrine neoplasia, type 2 A/B, familial medullary thyroid carcinoma, Pheochromocytoma** | High or near-complete penetrance | 171400 | *RET* | AD | Childhood/Adulthood |  |
|  |  | 162300 |  |  |  |  |
|  |  | 155240 |  |  |  |  |
|  |  | 171300 |  |  |  |  |
| **MUTYH-associated polyposis** | High or near complete penetrance | 608456 | *MUTYH* | AR | Adulthood |  |
| **Neurofibromatosis, type 2** | High or near complete penetrance  Age at onset can vary with variant type | 101100 | *NF2* | AD | Childhood/Adulthood |  |
| **Peutz-Jeghers syndrome** | High or near-complete penetrance | 175200 | *STK11* | AD | Childhood/Adulthood |  |
| **PTEN hamartoma tumor syndrome** | High or near-complete penetrance | 153480 | *PTEN* | AD | Childhood/Adulthood |  |
| **Von Hippel-Lindau syndrome** | High or near-complete penetrance.  Almost all individuals are symptomatic by age 65 years | 193300 | *VHL* | AD | Childhood/Adulthood |  |

^§^Actionable gene list as reported by Green RC, et al. Genet Med. 2013;15(7):565-74

AD, Autosomal dominant; AR, autosomal recessive; XL, X-linked; SD, semidominant
